# Voices of the Future: Junior Physicians’ Experiences of Discussing Life-Sustaining Treatments With Hospitalized Patients

**DOI:** 10.1177/23821205241277334

**Published:** 2024-09-05

**Authors:** Michael Andreas Müller, Claudia Gamondi, Eve Rubli Truchard, Anca-Cristina Sterie

**Affiliations:** 1 Palliative and Supportivecare Service, Lausanne University Hospital and University of Lausanne, Lausanne, Switzerland; 2 Service of Geriatrics and Geriatric Rehabilitation, Lausanne University Hospital and University of Lausanne, Lausanne, Switzerland; 3 Chair of Geriatric Palliativecare, Service of Palliative and Supportive Care and Service of Geriatrics and Geriatric Rehabilitation, Lausanne University Hospital and University of Lausanne, Lausanne, Switzerland

**Keywords:** Life-sustaining treatments, communication, junior physicians

## Abstract

**OBJECTIVES:**

Life-sustaining treatments (LST) aim to prolong life without reversing the underlying medical condition. Being associated with a high risk of developing unwanted adverse outcomes, decisions about LST are routinely discussed with patients at hospital admission, particularly when it comes to cardiopulmonary resuscitation. Physicians may encounter many challenges when enforcing shared decision-making in this domain. In this study, we map out how junior physicians in Southern Switzerland refer to their experiences when conducting LST discussions with hospitalized patients and their learning strategies related to this.

**METHODS:**

In this qualitative exploratory study, we conducted semi-directive interviews with junior physicians working at the regional public hospital in Southern Switzerland and analyzed them with an inductive thematic analysis.

**RESULTS:**

Nine physicians participated. We identified 3 themes: emotional burden, learning strategies and practices for conducting discussions. Participants reported feeling unprepared and often distressed when discussing LST with patients. Factors associated with emotional burden were related to the context and to how physicians developed and managed their emotions. Participants signaled having received insufficient education to prepare for discussing LST. They reported learning to discuss LST essentially through trial and error but particularly appreciated the possibility of mentoring and experiential training. Explanations that physicians gave about LST took into account patients’ frequent misconceptions. Physicians reported feeling under pressure to ensure that decisions documented were medically indicated and being more at ease when patients decided by themselves to limit treatments. Communication was deemed as an important skill.

**CONCLUSIONS:**

Junior physicians experienced conducting LST discussions as challenging and felt caught between advocating for medically relevant decisions and respecting patients’ autonomy. Participants reported a substantive emotional burden and feeling unprepared for this task, essentially because of a lack of adequate training. Interventions aiming to ameliorate junior physicians’ competency in discussing LST can positively affect their personal experiences and decisional outcomes.

## Introduction

Life-sustaining treatments (LST) are interventions whose aim is to prolong life without reversing the underlying medical condition; they include, among the most popularly known, the use of mechanical ventilation and cardiopulmonary resuscitation (CPR) but also others such as dialysis, artificial nutrition and hydration.^1^ Among LST, a particular interest is bestowed upon discussing and deciding whether CPR is medically indicated and desired by patients. Because CPR is associated with a high risk of developing unwanted adverse outcomes, such as frailty and neurological and physical disability,^2^ decisions about whether it should be attempted are routinely discussed with patients at hospital admission and documented on their hospital charts.

According to the shared decision-making model,^3^ considered the gold standard for doctor–patient communication about treatments and interventions, decisions about whether LST are relevant should be based on medical indication and patient preference, expressed autonomously.^4^ This, however, has been described as challenging in practice^5,6^ and laden with ethical difficulties.^7^ Difficulties are described as being generated by the anticipatory nature of decisions, the hypothetical nature of the medical conditions warranting LST that might arise and the tension between respecting patients’ autonomy and deciding what is considered medically indicated.^6,8^ An additional challenge may arise because some professionals deem shared decision-making suitable only in scenarios where legitimate choices are available (ie, indicated). In instances where LST are not considered medically indicated or beneficial, discussions tend to focus on the notion that they should be withheld rather than presenting patients with a choice.^9^ In this context, many healthcare professionals, particularly those not involved in specialized end-of-life care, experience conversations about LST as emotionally challenging.^10,11^ Furthermore, discussing LST was shown to be particularly challenging for junior physicians, who are generally responsible for hospital admissions.^12–14^

When discussing LST, particularly the relevance of CPR, adequate training is key. Self-perceived competence for discussing end of life is generally low for resident physicians.^15^ Authors have highlighted the importance of benefiting from role models and experienced leadership, particularly when discussing CPR or do-not-attempt-resuscitation (DNAR) orders.^6,16^ Training residents in communication skills for conducting LST conversations leads to enhanced patient understanding of the procedure and is correlated with an increased disposition to decline it.^16^ Drawing on this, a diversity of educational interventions for discussing LST has been made available, which, however, essentially focus on communication about CPR.^17–21^

While the challenging nature of LST discussions is undisputed, and particularly ethical aspects thereof are well covered in the medical literature, junior physicians’ real-life experiences seem to be an under-investigated field. The meaning of being a junior physician conducting discussions on resuscitation code status is not well covered.

## Objective

In this study, we map out how junior physicians practicing in Southern Switzerland refer to their experiences of conducting LST discussions with hospitalized patients. A particular interest was devoted to how participants learned to conduct these discussions.

## Methods

This qualitative, observational study was conducted by M.A.M. in response to the requirement to obtain his MSc in Medicine from the Università Della Svizzera Italiana. This study employs thematic analysis to explore the experiences of junior physicians conducting LST discussions. We adhered to the Consolidated Criteria for Reporting Qualitative Research (COREQ) checklist^22^ to ensure comprehensive and transparent reporting (Supplemental Material). Thematic analysis allowed us to systematically identify, analyze and report patterns within the data, providing rich and detailed insights into the participants’ experiences.

### Population

We recruited junior physicians working at the biggest regional public hospital in the canton of Ticino, Switzerland (Ente Ospedaliero Cantonale). Inclusion criteria consisted of being a physician. Exclusion criteria consisted in holding a post-graduate medical diploma.

### Recruitment

We employed a purposive sample approach. Junior physicians were informed of the study via email by their thesis supervisor or the department heads of Oncology, Internal Medicine and Surgical of the Ente Ospedaliero Cantonale. Those interested to partake in the study contacted us. More participants were recruited using a snowball recruitment strategy.

### Consent

Participants were informed of the study in written and orally and signed an informed consent agreement, following the procedure required by the regional ethics committee. A written information sheet detailed the study's objective, procedures and its potential benefits and risks; an appended consent form contained the signatures of both researcher and participant. The information and the consent were confirmed orally, before the interview.

### Data collection

No relationship was established prior to study commencement between the interviewer (M.A.M.) and the participants. Semi-directive face-to-face interviews were conducted from May 2021 to February 2022 at participants’ choice in English or Italian, based on an interview grid developed by M.A.M., with the input of C.G. Participants were informed that M.A.M. was conducting the study as part of his MSc in Medicine. After literature analysis, the grid was created to explore participants’ experiences before, during and after the discussion of LST and their experiences and opinions about communication skills training on this topic. It was reviewed after 3 initial interviews, and no modifications were made. No field notes were made during or after the interview. All interviews were conducted on hospital premises, audio-recorded and transcribed directly into English by M.A.M. Translations were checked by C.G., proficient in both Italian and English; they were not returned to participants for comment or correction. Interviews were conducted by M.A.M., who introduced himself as a medical student.

### Data analysis

Personal information in the transcripts was erased and transcripts were analyzed by hand, using an inductive thematic analysis.^23^ Random extracts from each interview were inductively and blindly coded in parallel by M.A.M. (a medical MA student) and C.G. (palliative care physician and M.A.M.'s supervisor) to assure reliability and for teaching purposes; codes were compared and discussed, and dissensions were resolved. M.A.M. inductively coded all the transcripts and developed an individual codebook for each interview. In the end, codebooks were merged, and identical and similar codes were grouped together. Based on this final codebook, M.A.M. identified themes and subthemes. C.G. and A.C.S. (medical sociologist) checked the process of code agglutination and theme/subtheme development upon its completion. E.R.T. (geriatrician) provided input on the discussion and the implications regarding future training.

To ensure the trustworthiness of our findings, we employed several strategies. Random extracts from each interview were inductively and blindly coded in parallel by M.A.M. and C.G. to enhance inter-coder reliability. C.G. and A.C.S. reviewed the process of code agglutination and theme/subtheme development to ensure consistency and accuracy. Additionally, a reflexivity statement has been included to address potential researcher biases. These steps, along with triangulation and member checking, contribute to the credibility and reliability of our qualitative analysis.

## Results

Nine physicians participated (see Table 1 for sociodemographic details) from oncology, internal medicine and surgical departments of the hospital.

**Table 1.** Participants’ demographics.

**CHARACTERISTICS**	**NUMBER**
Gender	
Female	4
Male	5
Age	
33 and under	5
Over 33	4
Years of medical practice	
<3	1
3-5	5
>5	3
Country of prior medical studies	
Italy	6
Switzerland	1
Other	2
Language of the interview	
Italian	4
English	5

Most had between 3 and 5 years of medical practice, and most were already relatively experienced; this was partially explained by the fact that they had undergone studies in another country. Interviews lasted 54 min (mean).

Twelve types of interventions were discussed (Table 2). Three main themes were identified: emotional burden, learning by doing and facilitating good decisions (Table 3). Several subthemes were developed to enable a finely grained analysis. Quotes have been selected to represent and illustrate different experiences and opinions expressed by participants.

**Table 2.** Interventions.

**INTERVENTION**	**EXAMPLE**
Intubation	“A lot of them, and so this was in the French part, they were really open and 90 percent of the patients said ‘No, please do not resuscitate me. I do not want to be intubated’”
Parenteral nutrition	“I have discussed parenteral nutrition recently… So we discussed with her and her family whether to start a therapy, nutrition through the veins or not”
Resuscitation	The first thing for me is to understand what is the indication, the medical indication for this patient. If you have to resuscitate him or not resuscitate him. If it would be my, my father will I resuscitate him
Dialysis	I go to the patient to discuss if he wants to be resuscitated if he wants to be dialyzed or UHM intubated and…
Antibiotics	Rational make me understand that it's useless to give antibiotics to this person UHM emotional it's another stuff
Sedation	But sometimes to make them feel better is the same as sedation. You have to… It's very difficult. But it's this process. Sedation is the right therapy for this patient
Chest compressions	I ask everyone what they want to do if the UHM the situation is going badly or if the heart stops or for like… the chest compression or if their lungs are not working, for intubation, for example
Going to intensive care	And he told me “I want to be resuscitated” and then I asked him specifically, for example for intensive care. P5 It is very difficult for a relative to understand, but also for us, on the other hand, to understand what will happen in intensive care. P8
Vasopressors	What does it mean to be under vasopressor, for example
Tube feeding	… if they want to continue to have tube feeding
Non-invasive ventilation	… she had a very, very worsening of her neurological status and respiratory status and then we decided not to anymore UHM non-invasive ventilation
Giving liquids	…and to have need of liquids…

**Table 3.** Themes, subthemes, and associated quotes.

**THEME (T)**	**SUBTHEME (ST)**	**QUOTES**
T1. Emotional burden	ST1. Timing/circumstances	“… I feel very uncomfortable because … there is a bit of pressure from the system, from the nurses asking you ‘Yes, or no?’.” (P2)
	ST2. Discussing DNR orders and life-prolonging measures	“… like how to discuss resuscitation … So maybe they make me uncomfortable, I shouldn't address these topics.” (P3)
	ST3. Lack of peer support	“… I thought we shouldn’t do it, because it was doing more harm than good … it wasn’t my decision. I still had to work with the patient and my colleagues. So, it was uncomfortable.” (P2)
	ST4. Developing personal sympathies	“He looked like my father, so it was a really …ehm…personal situation.” (P2)
	ST5. Feeling unable to provide adequate care	“… to see the patient that's quite cursed in their situation … that makes me feel sad in some ways.” (P5)
	ST6. Feeling unable to deal with patients’ reactions	But in medicine, many patients say to you: “But why are you asking me these things?” They almost get angry. … "Do you already give me up for dead?” (P8)
	ST7. Managing own's emotions	“… what happens here stays inside you. … And I carry it within me as an element that also built my personality.” (P9)
	ST8. Patients who can’t express themselves openly	“Many patients cannot express truly themselves or they are afraid sometimes to express truly themselves because they think they are going to be judged by their family, for example” P5
T2. Learning strategies	ST1. Insufficient training	“Not of a professional nature. In the sense that I never took a course … in the university period.” P7 “Why don't we do a course that gives us … an indication of how it could be done and especially what the goals are to achieve …” P8
	ST2. Observations	You have to look the other, more experienced… colleagues. P4
	ST3. Superior support	“…we prepared together before meeting the family members and patients for the discussion, and I did it in the presence of my superiors” P3
	ST4. Experience	“But if you experience something, it stays with you. … your brain or heart is so well trained, that it's kind of, the words come without even thinking.” P1
	ST5. Reading	“It's not at all like medical school or the books you are reading. You read them, you try to memorize and then definitely you will forget, if you don’t practice them.” P1
	ST6. Workshops	“So why don't we have a course that gives us, I don't want to say the tools, but that gives us a direction. SHORT PAUSE. OK. An indication of what it could look like, how it could be done and above all what the goals are to be achieved.” P8
	ST7. Inherent qualities	“Now as far as I'm concerned EHM fortunately, you know… It's kind of, part of… my attribute probably, which I consider at least an attribute, is the fact that I'm quite good with words.” P8
	ST8. Impact of COVID-19	“It was as if death was something… that in the moment it comes up, we decide. COVID however has … revealed this topic.” P9
T3. Practices	ST1. Timing and reason	“This is usually done with patients in whom one already knows that something serious will eventually happen.” P8 “… I feel a lot of discomfort because … there is a bit of the pressure from the system … you have to put a cross on a paper …” P9
	ST2. Initiation	“The first stuff, when you have decided what to do with the patient, you can go to him or to his UHM entourage and talk about, and I personally start with the summary” P4
	ST3. Explanation	“Show them all the various possibilities so that the patient can then choose.” P9
	ST4. Dealing with patient preference	“In these latitudes, there's still a bit of a do-it-all-for-all mentality.” P9 “They don’t understand what we talk about … We have to protect them … from the attachment to life” P4 “I feel a sense of relief because it is not me who has to decide, to choose, I can limit myself to respecting the patient's wishes.”P7
	ST5. Communication	“… I believe that we have to guide the patient in the decision.” P4 “We have responsibility… I don’t want to say the power… But somehow, we can guide the discussion. We can make it easier to accept one direction or another.” P6

### Theme 1. Emotional burden

Most participants reported an emotional burden when confronted with the obligation to discuss LST with patients newly hospitalized. Participants highlighted several factors contributing to the burden, which we organized into 9 subthemes.

Participants frequently reported the necessity to engage in these discussions at inopportune times and under unfavorable circumstances, such as in noisy and stressful environments (eg, the emergency room) and when patients are unprepared or not expecting to broach such a topic. This contingency contributed to creating an anxiogenic environment and emotional load. They also found that patients often needed more time to engage in these conversations, leading physicians to decide in their stead. This, in turn, was experienced as a burden. Institutional policies and pressures were considered as partially contributing to this (needing to document a decision as soon as a patient is admitted).

The topic itself, often associated with death, was felt as irrelevant to be addressed in the context of hospitalization, when the expected outcome is being treated and recovering health.

Another factor concerned the lack of peer support. Participants evoked the need to deal with disagreements from hierarchic superiors or colleagues, being forced to provide treatment that they disagree with and not benefiting from the support of specialists when called to disclose a bad prognosis.

Emotional burden was also triggered by participants’ developing personal sympathies with a patient, which made physicians “feel sorry for” them, as well as by feeling unable to provide adequate care, when not prescribing certain treatments and interventions that could extend life, even though at a low quality.

Participants also had a difficult time dealing with emotions. Firstly, this concerned patients’ and relatives’ reactions. In general, participants feared that patients would find the discussions unpleasant. One participant felt that his lack of competence in this domain was due to his profession and thought spiritual consultants might be better prepared to handle this aspect of patient care. Also, participants expressed difficulties managing their own negative emotions generated by these discussions, for example, feeling like “the bad guy,” even when the medical decision was clinically “correct” because of depriving patients or relatives of hope. One participant hypothesized that all physicians might experience a duality between emotions and rational decision-making. Another referred to the need to seem “rational” and hide emotions in front of patients. According to the participants, repeated exposure to these experiences can contribute to better management of emotions but might also lead to desensitization, for example, losing empathy. Related to this, participants also identified dealing with memories of difficult conversations that persist as “a baggage that maybe is not going to leave you,” leading to overthinking and ruminations.

A final factor concerned situations when participants felt that patients could not express themselves openly during a discussion, for example, due to family pressure.

### Theme 2. Learning strategies

Participants openly shared experiences and opinions about adequate learning strategies that prepare junior physicians to discuss LST with hospitalized patients. We identified 9 subthemes.

A majority of participants considered that they received insufficient university training in this domain, with missing courses or courses being too short and informal. Participants felt that they developed their communication skills while practicing medicine as junior physicians and were desireful of more intense and adapted courses on discussing LST in the undergraduate curricula, as other clinical skills.

Participants referenced a range of learning strategies, either drawn from personal experiences or aspired to, including observing more experienced physicians, seeking support from superiors through measures like devising a discussion strategy before patient meetings, progressing through personal experience, experimenting with various methods and learning from mistakes, albeit acknowledging the time investment required for these insights to become ingrained. Contrarily, reading was not deemed a particularly relevant resource in this domain. However, experiential workshops received more favorable evaluations. While some participants believed that no specific methods could aid in acquiring the skills for conducting LST conversations, they asserted that success was contingent on inherent characteristics of individual doctors who possessed a natural “gift” for effective patient communication.

Lastly, participants referred to how COVID-19 impacted their learning of conducting LST conversations. Participants remarked that the COVID-19 crisis was an opportunity to practice their skills further since LST conversations, in particular about CPR, were even more important and, at the same time, more difficult (eg, because they took place when patients’ health was critical and in conditions in which resources were upon constraint).

### Theme 3. Practices for discussing LST

Participants frequently referred to their practices for discussing LST with patients throughout their interviews. We condensed the characteristics of these practices in 6 subthemes.

The first aspect describing practices consisted of when and why LST discussions are initiated, rooted in either institutional policies (the way things are “usually done,” requested by the hospital policy or expected by colleagues) or medical necessity (patient's health status).

Regarding how discussions are initiated, participants often referred to summarizing the situation, which also justifies its reason. Participants would then offer explanations about the procedure, including CPR, intubation, the use of antibiotics and the patient's condition. Most participants convened that patients often had scarce or inaccurate information about their condition, influenced by exposure to media but also bound to the cultural context and that this would weigh in on their decisions. In particular, one participant referred to the fact that he explained the technicalities and consequences of attending intensive care to correct patients’ expectations regarding their benefits.

When dealing with patient preference, participants often referred to cases when patients select extreme measures that would not be beneficial and to their duty to protect patients from this tendency and some of them, to the need to decide against them. Several participants oriented towards the gold standard of shared decision-making and respecting patient autonomy and expressed being distressed when this couldn’t be achieved. Physicians reported feeling more at ease with respecting patients’ choices when they decide of their own volition to limit treatments and not undergo CPR and less responsible for the decision made. All throughout, participants referred to the need and pressure to ensure that decisions are medically indicated.

Communication with patients and having a “good discussion” were considered an important skill. At the same time, it was also highlighted as a resource in convincing patients to select certain courses of action, for example, by way of presenting and describing options of care and the medical recommendation. One participant referred to practices of not having in-depth conversations with patients about the possibility of not undergoing CPR. Relatives’ and patients’ educational levels were highlighted as barriers to discussions and causing discomfort to the participants.

## Discussion

Discussions about whether LST, especially CPR, should be attempted are routine at hospital admissions. In this exploratory study, we conducted an inductive thematic analysis to map out how junior physicians practicing in Southern Switzerland experience these conversations. We developed 3 overarching themes for aspects participants referred to in their interviews: the emotional burden they experience, the learning strategies and their practices for conducting discussions.

In spite of the routinized character of the discussion, many physicians, especially junior ones,^12–14^ feel unprepared and often distressed to discuss DNAR with their patients.^5,6,24^ This reality is well reflected in our data, with the context being identified as one of the essential factors leading to distress, particularly the institutional policies that participants felt pressured to comply with even when these discussions were judged inappropriate, as well as the lack of peer support. While prior literature specifically refers to the difficulty of discussing cases of medical futility,^25^ our participants also referred to the content of the discussion (discussing DNAR, CPR and generally LST). Another context-related factor leading to distress was the lack of support. Other factors were physician-related, essentially related to how participants developed and managed (or not) their emotions. As prior literature highlighted,^26^ intrinsic factors, such as death anxiety, bear a non-negligible weight on how these conversations are experienced.

The role of adequate training and education in junior physician's preparedness and competence in leading conversations about DNAR with patients is evident.^6,15,16^ In our data as well, all participants signaled not having benefitted from sufficient education to prepare for discussing LST. However, since they had a median of 5 years of medical practice, this shows that time in practice alone does not guarantee sufficient training in this matter. As Levine et al^27^ showed, often existent training programs are not accessible before advanced residency. Our participants referred to various strategies for gaining competence, of which the most important seemed to be the observation of more experienced colleagues, ie, role modelling or mentoring^28–30^ and support from their superiors. The gap between undergraduate learning and practice was evident, as noted in other studies.^31^ Participants favored communication skills workshops more than theoretical-based learning. However, participants mostly learned to discuss DNAR orders on the job through trial and error.

When it comes to practices through which participants conduct LST discussions, they emphasized the importance of preserving the patient's autonomy by favoring shared decision-making, respecting patients’ choices and highlighting the need for decisions to be medically indicated. This ambivalence was highlighted in other studies.^32,33^ The tension between these 2 goals was manifest and often gave way to complex emotions arising from these conversations. Participants explained that they often made unilateral decisions on resuscitation if they thought it was contraindicated. By phrasing questions and portraying specific options in a particular way, they tried to lead their patients towards the decision they thought was right for them. This finding is in line with the qualitative study by Schoenfeld et al,^34^ which identified the application of “guided shared decision-making” in cases where physicians intended what decisions patients should make. Some participants reported being conscious about their power to influence patients’ decision-making. This finding broadly corroborates the study by Dzeng,^35^ which reported that physicians were conflicted between being neutral conveyors of information and convincing “the decision-maker to pursue the right treatment.”

### Strengths and limitations

This study comprises an in-depth investigation of how junior physicians discuss LST with patients. While this topic has received much attention lately, it has mainly focused on experiences surrounding conversations about CPR and DNAR, without considering LST in general. Also, participants’ experience in Southern Switzerland has never been investigated before. Through utilizing the interview grid with open questions, participants were free to answer and raise topics they thought were important. Their responses often converged, thus indicating the potentiality of representativeness. However, we acknowledge that the majority of participants had undergone their undergraduate education off broad, and the barriers they report in this regard may not represent the educational situation in Switzerland. In retrospective, we also acknowledge that the interview grid might have directed participants to emphasize factors influencing their experiences rather than describing their feelings and emotions in detail. Overall, responses and experiences focused on CPR, and though LST was referred to as a category, other treatments except CPR were rarely referred to explicitly. Furthermore, we realize that participants knew this study were supervised by a senior physician in the Ente Ospedaliero Cantonale. Even though participants were assured of anonymity, social desirability might have played a role in the accounts given during the interviews. More participants would have allowed to reach data saturation.

## Conclusion

Junior physicians in our study reported that conducting LST discussions was a challenging task, in particular when needing to talk about CPR, and that they felt caught between counselling patients to make the right decisions and respecting patients’ autonomy in decision-making. They also reported experiencing a substantive emotional burden and feeling unprepared for this task because of a lack of adequate formal, pre- and post-graduate training. In their practice, they mostly rely on role modelling, supervision and personal experience as learning methods, which is not sufficient as a basis for learning.

Interventions aiming to improve junior physicians’ competency in discussing LST, particularly CPR orders, might positively affect their personal experiences as well as those of the patients and the outcomes of the discussion. In-depth academic education on communication skills and conducting difficult conversations may enhance technical capacities to conduct these difficult conversations, possibly reducing the emotional burden and the trial-and-error learning model. Education can also enhance personal confidence in conducting such discussions. Formal programs could take up the learning methods that are used and ensure that they are employed in a controlled manner. Further research could explore the learning methods used by junior physicians in more depth. Additional research is needed to characterize the tension participants felt between the autonomy of patients and their intention to minimize suffering and avoid futile treatments. Future research could also shed light on how junior physicians reflect upon their values and judge what they hold dear and how they want to practice medicine.

### Reflexivity statement

The author conducting the interviews and leading the analysis identified the topic following a personal family experience with curative treatment of patients nearing the end of life. This personal experience, combined with the author's professional background as a physician in training, necessitated careful consideration of how personal beliefs might influence the construction of the interview guide and the management of interview topics. Face-to-face contact during the interviews required the author to remain aware of these potential biases and strive for objectivity throughout the research process

## Supplemental Material

sj-doc-1-mde-10.1177_23821205241277334 - Supplemental material for Voices of the Future: Junior Physicians’ Experiences of Discussing Life-Sustaining Treatments With Hospitalized Patients Supplemental material, sj-doc-1-mde-10.1177_23821205241277334 for Voices of the Future: Junior Physicians’ Experiences of Discussing Life-Sustaining Treatments With Hospitalized Patients by Michael Andreas Müller, Claudia Gamondi, Eve Rubli Truchard and Anca-Cristina Sterie in Journal of Medical Education and Curricular Development
